# Assessing genotype–phenotype correlations in colorectal cancer with deep learning: a multicentre cohort study

**DOI:** 10.1016/j.landig.2025.100891

**Published:** 2025-08-19

**Authors:** Marco Gustav, Marko van Treeck, Nic G Reitsam, Zunamys I Carrero, Chiara M L Loeffler, Asier Rabasco Meneghetti, Bruno Märkl, Lisa A Boardman, Amy J French, Ellen L Goode, Andrea Gsur, Stefanie Brezina, Marc J Gunter, Neil Murphy, Pia Hönscheid, Christian Sperling, Sebastian Foersch, Robert Steinfelder, Tabitha Harrison, Ulrike Peters, Amanda Phipps, Jakob Nikolas Kather

**Affiliations:** Else Kroener Fresenius Center for Digital Health (M Gustav MSc, M van Treeck MSc, Z I Carrero PhD, C M L Loeffler MD, A Rabasco Meneghetti PhD, Prof J N Kather MD) and Department of Medicine I (C M L Loeffler, Prof J N Kather), Faculty of Medicine and University Hospital Carl Gustav Carus, Dresden University of Technology, Dresden, Germany; Pathology, Faculty of Medicine, University of Augsburg, Augsburg, Germany (N G Reitsam MD, Prof B Märkl MD); Division of Gastroenterology and Hepatology, Mayo Clinic, Rochester, MN, USA (Prof L A Boardman MD); Division of Laboratory Genetics, Department of Laboratory Medicine and Pathology, Mayo Clinic, Rochester, MN, USA (A J French MSc); Department of Quantitative Health Sciences, Division of Epidemiology, Mayo Clinic, Rochester, MN, USA (Prof E L Goode PhD); Center for Cancer Research, Medical University of Vienna, Vienna, Austria (A Gsur PhD, S Brezina PhD); Nutrition and Metabolism Branch, International Agency for Research on Cancer, WHO, Lyon, France (Prof M J Gunter PhD, N Murphy PhD); Cancer Epidemiology and Prevention Research Unit, School of Public Health, Imperial College London, London, UK (Prof M J Gunter); Institute of Pathology, University Hospital Carl Gustav Carus, Dresden University of Technology, Dresden, Germany (P Hönscheid PhD, C Sperling MTLA); National Center for Tumor Diseases, Partner Site Dresden, German Cancer Research Center Heidelberg, Dresden, Germany (P Hönscheid); German Cancer Consortium and German Cancer Research Center, Heidelberg, Germany (P Hönscheid); Institute of Pathology, University Medical Center Mainz, Mainz, Germany (S Foersch MD); Division of Public Health Sciences, Fred Hutchinson Cancer Center, Seattle, WA, USA (R Steinfelder PhD, T Harrison MPH, Prof U Peters PhD, A Phipps PhD); Department of Epidemiology, University of Washington, Seattle, WA, USA (T Harrison, Prof U Peters, A Phipps); Medical Oncology, National Center for Tumor Diseases, University Hospital Heidelberg, Heidelberg, Germany (Prof J N Kather); Pathology and Data Analytics, Leeds Institute of Medical Research at St James’s, University of Leeds, Leeds, UK (Prof J N Kather)

## Abstract

**Background:**

Deep learning-based models enable the prediction of molecular biomarkers from histopathology slides of colorectal cancer stained with haematoxylin and eosin; however, few studies have assessed prediction targets beyond microsatellite instability (MSI), *BRAF*, and *KRAS* systematically. We aimed to develop and validate a multi-target model based on deep learning for the simultaneous prediction of numerous genetic alterations and their associated phenotypes in colorectal cancer.

**Methods:**

In this multicentre cohort study, tissue samples from patients with colorectal cancer were obtained by surgical resection and stained with haematoxylin and eosin. These samples were then digitised into whole-slide images and used to train and test a transformer-based deep learning algorithm for biomarker detection to simultaneously predict multiple genetic alterations and provide heatmap explanations. The primary dataset comprised 1376 patients from five cohorts who underwent comprehensive panel sequencing, with an additional 536 patients from two public datasets for validation. We compared the model’s performance against conventional single-target models and examined the co-occurrence of alterations and shared morphology.

**Findings:**

The multi-target model was able to predict numerous biomarkers from pathology slides, matching and partly exceeding single-target transformers. In the primary external validation cohorts, mean area under the receiver operating characteristic curve (AUROC) for the multi-target transformer was 0⋅78 (SD 0⋅01) for *BRAF*, 0⋅88 (0⋅01) for hypermutation, 0⋅93 (0⋅01) for MSI, and 0⋅86 (0⋅01) for *RNF43*; predictive performance was consistent across metrics and supported by co-occurrence analyses. However, biomarkers with high AUROCs largely correlated with MSI, with model predictions depending considerably on morphology associated with MSI at pathological examination.

**Interpretation:**

By use of morphology associated with MSI and more subtle biomarker-specific patterns within a shared phenotype, the multi-target transformers efficiently predicted biomarker status for diverse genetic alterations in colorectal cancer from slides stained with haematoxylin and eosin. These results highlight the importance of considering mutational co-occurrence and common morphology in biomarker research based on deep learning. Our validated and scalable model could support extension to other cancers and large, diverse cohorts, potentially facilitating cost-effective pre-screening and streamlined diagnostics in precision oncology.

**Funding:**

German Federal Ministry of Health, Max-Eder-Programme of German Cancer Aid, German Federal Ministry of Education and Research, German Academic Exchange Service, and the EU.

## Introduction

Exome and targeted panel sequencing are central to precision oncology of colorectal cancer, but remain inaccessible to many patients worldwide due to expensive equipment and complex data analysis.^1^ By contrast, histopathology slides stained with haematoxylin and eosin are standard diagnostic tools used globally. The advent of deep learning has revealed these slides as a quantifiable data resource. Studies published over the past several years show that deep learning can predict molecular biomarkers directly from digitised slides stained with haematoxylin and eosin, including microsatellite instability (MSI),^2–6^ hypermutation, and gene mutations such as *TP53*, *BRAF*, and *KRAS.*^3–10^ When applied as pre-screening tools, these systems based on deep learning can streamline the diagnostic workflow, identifying cases that need further testing and ruling out others.^11^

Previous studies on deep learning in colorectal cancer have predominantly targeted specific genetic alterations as potential biomarkers, referred to as prediction targets in computational pathology, with limited adoption of panmolecular frameworks.^7,10^ These approaches are constrained by the scarcity of comprehensive, multicohort sequencing datasets and the need to train separate models for each target,^3,4,8,9^ rendering them labourintensive and resource-intensive. Addressing these limitations, this study introduces, to our knowledge, the first approach based on pan-biomarker deep learning for colorectal cancer by use of a multicentre cohort, applying a single transformer-based model to simultaneously predict multiple molecular targets.

## Methods

### Study design

In this multicentre cohort study, we developed a deep learning model using a single transformer model to predict multiple genetic alterations directly from anonymised slides stained with haematoxylin and eosin of colorectal cancer. The model was trained and validated on a dataset from the Genetics and Epidemiology of Colorectal Cancer Consortium (GECCO), which aggregates sequencing data across diverse cohorts.^12^ Generalisability was assessed in two external cohorts of patients with colorectal cancer.

Given the robust performance of deep learning in linking phenotype to genotype, particularly for the prediction of MSI,^3,4^ we extended this analysis to multiple prediction targets using our comprehensive dataset and developed model. We assessed the co-occurrence of genetic alterations with MSI, evaluated their predictability based on deep learning, and examined corresponding slide morphology in relation to features linked to MSI. Prediction targets encompassed both literature-reported alterations^3–10^ and clinically relevant genes (ie, *BMPR2*, *RNF43*, and *BRAF)*, as well as MSI and hypermutation.

This study is reported according to TRIPOD guidelines and was performed in accordance with the Declaration of Helsinki. All cohorts contributing to GECCO obtained written informed consent from all participants and received approval from their respective institutional review boards. The harmonised, de-identified genotype–phenotype data analysed in this Article were accessed via the NIH Database of Genotypes and Phenotypes (under study accessions phs001078.v1.p1 and phs001415.v1.p1). For each individual study, we have added the reference with details available in the appendix (pp 2–3). The overall analysis was approved by the ethics board of the Medical Faculty of Technical University Dresden (ID BO-EK-444102022).

### Cohorts and sequencing

**Figure 1:**
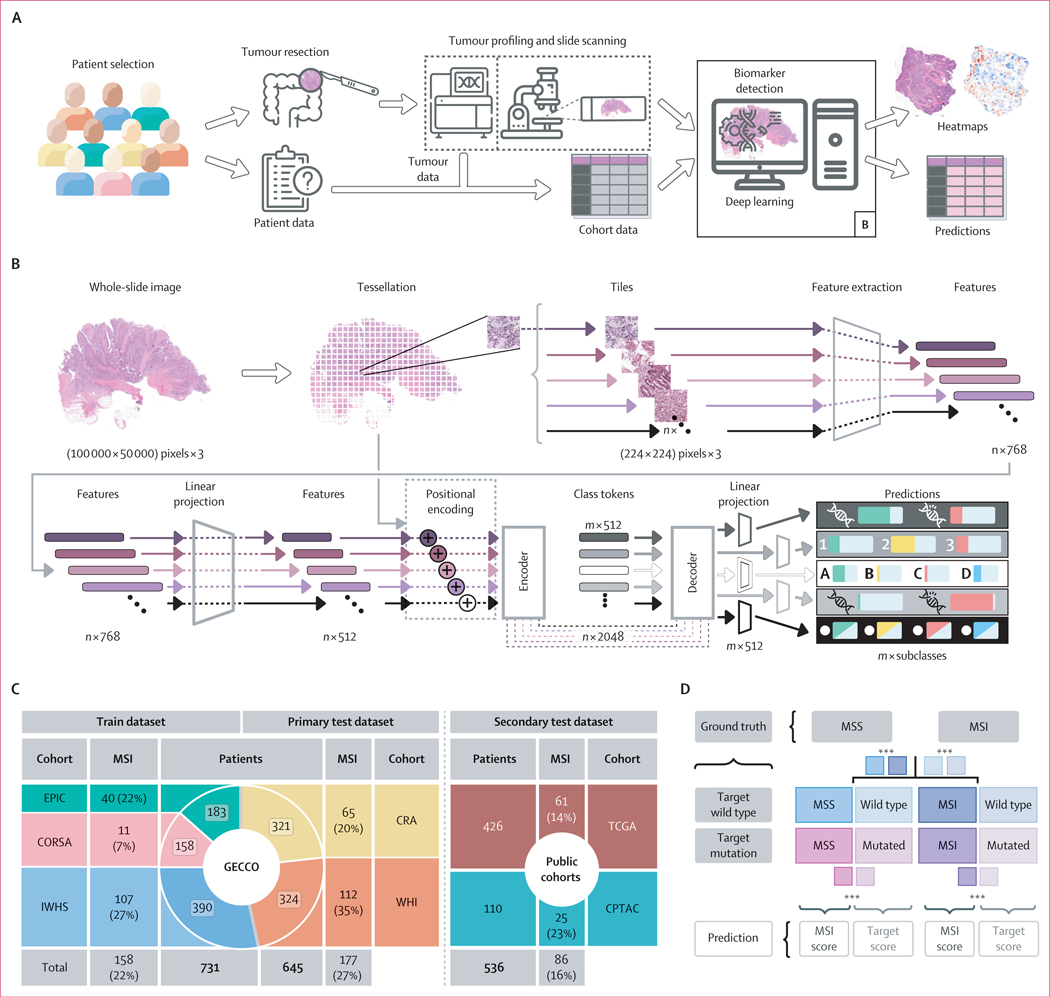
Experimental design, cohort characterisation, and schematic for predictive analysis (A) Tissue samples from patients with colorectal cancer across five independent cohorts were obtained by surgical resection, and associated demographic, clinical, and sequencing data were collected. Following staining with haematoxylin and eosin, tumour tissues were digitised into whole-slide images for profiling genetic alterations. The whole-slide images were then used to train and test a deep learning-based algorithm for biomarker detection to simultaneously predict multiple mutational statuses and provide heatmap explanations. (B) The deep learning-based pipeline tessellated the whole-slide images into smaller tiles while rejecting background and blurry areas, extracting feature vectors from tiles. Feature vectors were compressed and processed in a multi-target transformer, applying an attention mechanism in an encoder–decoder structure for class token learning. The transformer generated individual scores for the respective number of classes per target. The code was able to comprise positional tile embedding (dashed lines), which did not result in improved performance and was therefore excluded from the study. Numbers (eg, *n* × 768) denote matrices, where *n* is the number of extracted image tiles and 768 is the length of each tile’s feature vector. Subsequent steps transform these to different dimensions (eg, *n* × 512). *m* × 512 refers to class token representations (*m* class tokens representing *m* prediction targets), and *m* × subclasses refer to the final prediction outputs for each class or subclass. (C) Overview of the five GECCO and two public cohorts, including patient numbers, slides, extracted features, and proportions of MSI cases. The GECCO cohorts were divided into train datasets and primary test datasets. (D) Schematic for interpreting result plots and statistics, delineating dataset partitioning based on microsatellite status (MSS or MSI) and mutational status (mutated or wild type). The diagram illustrates distinct groups by colour, with the left side representing MSI prediction scores and the right side showing prediction target scores. Ground truth labels of samples guide the group organisation, with model-generated scores depicted in corresponding colours. CORSA=Colorectal Cancer Study of Austria. CPTAC=Clinical Proteomic Tumor Analysis Consortium. CRA=Cancer Risk Assessment study. EPIC=European Prospective Investigation into Cancer. GECCO=Genetics and Epidemiology of Colorectal Cancer Consortium. IWHS=Iowa Women’s Health Study. MSI=microsatellite instability. MSS=microsatellite stability. TCGA=The Cancer Genome Atlas. WHI=Women’s Health Initiative.

The primary dataset comprised 1376 patients with colorectal cancer from five GECCO^12^ cohorts: European Prospective Investigation into Cancer (EPIC; 183 [13⋅3%]), Colorectal Cancer Study of Austria (CORSA; 158 [11⋅5%]), Iowa Women’s Health Study (IWHS; 390 [28⋅3%]), Cancer Risk Assessment study (CRA; 321 [23⋅3%]), and Women’s Health Initiative (WHI; 324 [23⋅5%]; figure 1A–C). Tissue samples were obtained from patients by surgical resection and stained with haematoxylin and eosin, before being digitised into whole-slide images. Besides whole-slide images, each cohort provided harmonised data on clinical features, demographics, and lifestyle (appendix pp 2–3).

Tissue samples underwent centralised targeted sequencing of up to 356 genes, with assessment of MSI and hypermutation status. The analysis focused on non-silent mutations and mutational signatures with a panel of 1⋅8 megabases, with tumour coverage of 975× and normal coverage of 273×.

To assess generalisability, we used two external secondary datasets on 536 patients with colorectal cancer with whole-slide images and matched molecular profiles (figure 1C). The first dataset was The Cancer Genome Atlas (TCGA) comprising samples from 426 patients,^13^ with MSI defined per Liu and colleagues:^14^ high MSI was categorised as MSI, and low MSI and microsatellite stability (MSS) were categorised as MSS.^5^ The second dataset was the Clinical Proteomic Tumor Analysis Consortium (CPTAC) comprising fresh-frozen colon tissue samples from 110 patients.^15,16^

In total, 1912 participants were included across all seven cohorts in the primary and secondary datasets, of whom 1290 (67⋅5%) were women and 621 (32⋅5%) were men.

Most patients were White (1449 [75⋅8%]), with smaller proportions identifying as Black (82 [4⋅3%]), Asian (33 [1⋅7%]), and American Indian or Alaska Native (three [0⋅2%]; appendix pp 2–3). Molecular annotations were obtained from the Genomic Data Commons Data Portal. All collected data were anonymised.

### Experimental design

The prediction panel comprised alterations previously assessed by deep learning, clinically relevant mutations, and genes strongly associated with MSI in the TCGA cohort, including *APC*, *BMPR2*, *BRAF*, *KRAS*, *RNF43*, *TP53*, and *ZNRF3*,^3,4,7–9,17^ as well as MSI, hypermutation, and additional targets from the GECCO cohorts (appendix pp 4–6). Continuous prediction targets were discretised at pre-set thresholds for balanced class distribution; targets with fewer than 20 cases per class were excluded to ensure robustness.

Model training involved 731 patients from the EPIC, CORSA, and IWHS cohorts (figure 1C), in which seven-fold cross-validation was used to balance training and validation sets and to identify a median-performing model. The resulting seven models were deployed on external test sets. The primary test set comprised 645 patients from the CRA and WHI cohorts, ensuring a representative split addressing missing cases, class distribution, and inclusion of all-women cohorts in both training (IWHS) and testing (WHI). Generalisability was evaluated on a secondary test set comprising the TCGA and CPTAC cohorts. External validation and test set refer to the primary test set unless stated otherwise; cohort-wise analyses are explicitly noted.

We conducted a total of 11 experiments using multi-target models trained on identical internal datasets: a primary model incorporating all targets (appendix pp 4–6), a secondary model excluding MSI to assess its impact, and nine single-target models (one per main alteration). Primary and secondary models were tested on both test sets; single-target models were evaluated on the primary test set. All models were compared with the primary model and literature benchmarks.

### Co-occurrence analysis

To investigate the co-occurrence of genetic alterations with MSI in colorectal cancer, we analysed alterations with complete data from the GECCO cohorts (appendix pp 2–3). We quantified pairwise relationships using a two-step procedure (appendix p 1). Briefly, we grouped mutations using hierarchical clustering, followed by association rule mining to identify co-occurrence patterns between mutations and clinical features, such as MSI. Rules were generated with an initiating genetic alteration (ie, antecedent) and a potentially resulting alteration (ie, consequent). The presence of an initiating alteration statistically increases the likelihood of observing a resulting alteration, without implying biological causation.

### Image processing and deep learning techniques

Digitised whole-slide images were tessellated into tiles of 224 × 224 pixels, corresponding to 256 × 256 μm (figure 1B).^5^ Tiles predominantly containing background (brightness value ≥224) or blur, defined by up to 2% edge pixels via Canny edge detection (thresholds 40–100),^18^ were discarded. A transformer-based^5^ encoder–decoder model for simultaneous multi-target prediction (figure 1B) was trained from scratch on the training set, by use of 768-dimensional tile-level embeddings extracted with the fixed, pre-trained CTransPath feature extractor,^19^ which was not fine-tuned during training. The tile embeddings were projected into a 512-dimensional space with a fully connected layer to reduce model complexity and improve computational efficiency.

Encoded tokens were decoded into 1 × 512 dimensional class tokens,^5^ each corresponding to a distinct prediction target. These tokens were passed through a final fully connected layer to generate target-specific predictions, with scores ranging from 0⋅00 (negative prediction or wild type) to 1⋅00 (positive prediction or mutated) per class and patient. To address class imbalance during training, cross-entropy loss was computed per target, weighted by the inverse mutation frequency, and summed to ensure proportional importance of rare mutations. Model training and inference were conducted on an NVIDIA RTX A6000 (48 GB memory). Links to the directories containing all source code used for the trained models are available in the appendix (p 7).

### Explainability

To investigate the model’s detection of relevant regions for alteration prediction, we generated heatmaps based on Grad-CAM^9^ using the fold with the median area under the receiver operating characteristic curve (AUROC) for the detection of MSI to ensure consistent comparisons. Heatmaps highlight the contribution of each tile to patient-level predictions, visualising the morphological features the model relies on. Notably, many positively contributing tiles do not necessarily yield a high final score due to non-linear aggregation and reliance on global cues not captured by the heatmap. We identified highly predictive top tiles for representative cases, selected on the basis of their scores and attention values assigned by the model, and included top tiles for each prediction target alongside those for MSI from the same slide. We also analysed class-token interactions in the decoder during deployment of the primary model to assess overlap for the main prediction targets. Grad-CAM was used to compute activations, capturing class token score interactions, which were aggregated into a cross-correlation matrix, averaged across patients, and visualised as a heatmap.

### Statistical analysis

Cohort characteristics are summarised with descriptive statistics (appendix pp 2–3). We assessed model performance on training and test sets using AUROC, complemented by additional metrics and target-specific prediction scores to provide a more representative evaluation in the context of class imbalance due to rare mutations. Two-sided DeLong tests were used to compare AUROCs between single-target and multi-target models on the primary test dataset, applying mean prediction scores across seven folds. Mean, median, and corresponding measures of variability in AUROCs were calculated per target across folds.

Additional DeLong tests compared the primary model (including MSI) with the secondary model (excluding MSI) within each of the four external cohorts, based on mean scores from the seven models. For each main prediction target, the mean score per patient across seven folds was stratified into the four following subgroups based on the patient’s microsatellite status and target mutation status: MSS and wild type, MSS and mutated, MSI and wild type, and MSI and mutated (figure 1D).

Normality was tested with the Shapiro–Wilk test; statistical significance between MSI and target scores within subgroups was assessed with Mann–Whitney tests. Wilcoxon tests evaluated model discrimination per target between wild type and mutated within MSS and MSI tumours (ie, MSS and wild type *vs* MSS and mutated; MSI and wild type *vs* MSI and mutated) and between microsatellite states within wild-type and mutated tumours (ie, MSS and wild type *vs* MSI and wild type; MSS and mutated *vs* MSI and mutated). All statistical analyses were performed in Python (version 3.11.9) and SciPy (version 1.14.0).

### Role of the funding source

The funders of the study had no role in study design, data collection, data analysis, data interpretation, or writing of the report.

## Results

**Figure 2:**
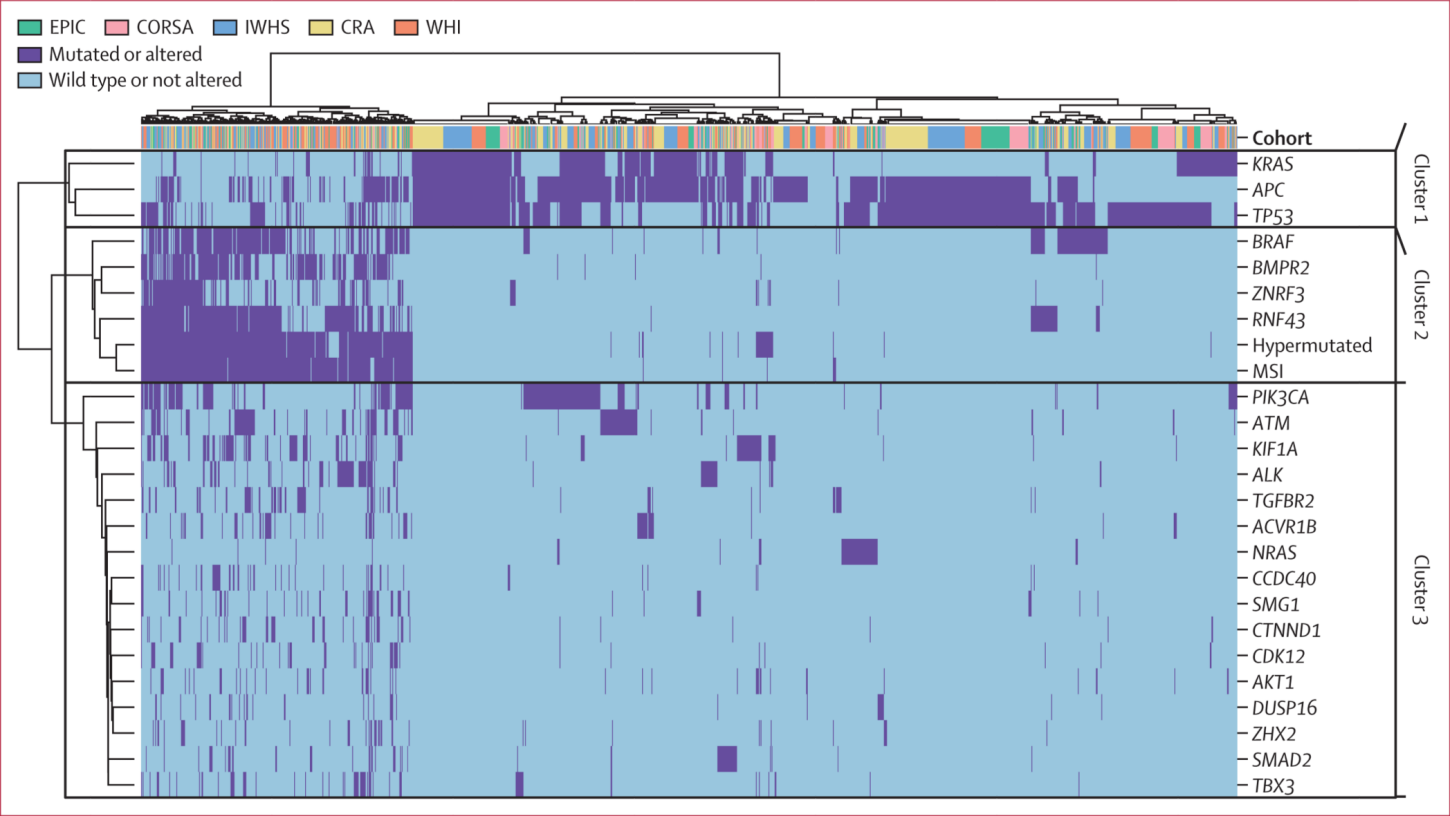
Analysis of the co-occurrence of genetic alterations in cases of colorectal cancer in the GECCO cohorts Hierarchical clustering analysis was conducted on the ground truth of genetic alterations with fully available information on mutational status. Each row corresponds to a genetic alteration and each column represents a patient from the dataset. The top row indicates the distribution of patients from various cohorts within genetic clusters. The distance calculation was performed with the Euclidean metric and the Ward method was applied to clustering (appendix p 1). Three unique genetic clusters were identified and are indicated by horizontal lines and labelled as Cluster 1, Cluster 2, and Cluster 3 along the right side of the heatmap. The patient clustering shows a diverse distribution of samples across all five cohorts and genetic clusters (top row). CORSA=Colorectal Cancer Study of Austria. CRA=Cancer Risk Assessment study. EPIC=European Prospective Investigation into Cancer. GECCO=Genetics and Epidemiology of Colorectal Cancer Consortium. IWHS=Iowa Women’s Health Study. WHI=Women’s Health Initiative.

Our approach aimed to reproduce the findings of previous deep learning studies that predicted genetic alterations in colorectal cancer from slides stained with haematoxylin and eosin,^2–4,7–9,20^ extending to a broader set of alterations through a multi-target transformer architecture (figure 1B). We compared the external validation performance of our transformer for selected targets, including main prediction targets (figure 2), with the external validation AUROCs reported in the literature for single-target models, which used varying model architectures.

**Figure 3:**
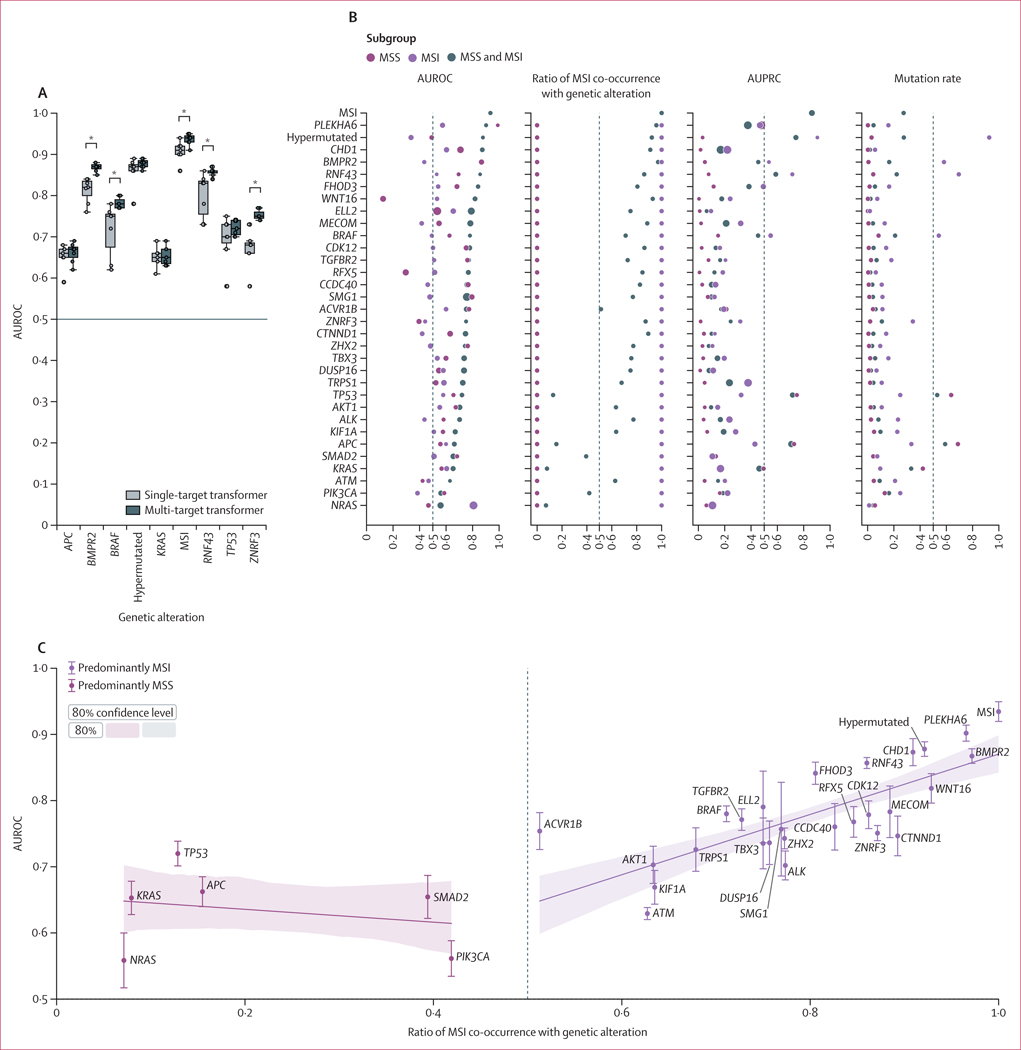
Evaluation of the performance of the multi-target transformer on selected prediction targets for the external cohorts from GECCO (A) The comparison between single-target transformer and multi-target transformers shows the AUROC from each of the seven folds of external cross-validation, with the median value highlighted with a horizontal line in each box. Selected representative potential biomarkers of genetic alterations associated with MSS and MSI are shown. The test set cohorts consist of CRA and WHI. The horizontal line positioned at an AUROC of 0⋅50 represents a random guess of the model. Significance was determined through a two-sided DeLong test with a p value threshold of less than 0⋅05, with * indicating p<0⋅05. (B) Performance metrics of multi-target transformers for external validation. The mean (centre of dot) and SD (diameter of dot) for relevant selected prediction targets for the external set, as well as the MSI and MSS subgroups, are shown based on the seven folds of cross-validation. The threshold for binary classification was predefined as 0⋅50. The evaluation metrics include the AUROC and AUPRC, along with the corresponding mutation rates in external cohorts. Mutation rate refers to the fraction of instances with a specific mutation in the subgroup. MSI and the ratio of genetic alteration co-occurrence is the fraction of cases with MSI among all cases with a particular genetic mutation. Performance metric data and an extended version of this panel with more metrics is shown in the appendix (pp 20, 26–27, 34–35). (C) Distribution of mean AUROCs (SD) for selected prediction targets and their co-occurrence with MSI; corresponding values and further metrics are shown in the appendix (p 20). An extended version of this panel with AUROCs specific to MSS and MSI subgroups is provided in the appendix (pp 34–35). AUPRC=area under the precision recall curve. AUROC=area under the receiver operating characteristic curve. CRA=Cancer Risk Assessment study. GECCO=Genetics and Epidemiology of Colorectal Cancer Consortium. MSI=microsatellite instability. MSS=microsatellite stability. WHI=Women’s Health Initiative.

For the detection of MSI, mean AUROC was 0⋅91 (SD 0⋅02) for single-target transformers and 0⋅93 (0⋅01) for multi-target transformers on the primary test set (p=0⋅0015; figure 3A; appendix p 8). Mean AUROCs for the primary model ranged from 0⋅87 (SD 0⋅01) in the TCGA cohort to 0⋅94 (0⋅01) in the WHI cohort (appendix pp 9–12, 31). For selected targets, such as the detection of a *BRAF* mutation, mean AUROC was 0⋅72 (SD 0⋅06) for the single-target transformer and 0⋅78 (0⋅01) for the multi-target trans-former (p<0⋅0001; figure 3A; appendix p 8). Mean AUROCs for *BRAF* in the primary model (multi-target transformer including MSI) versus the secondary model (multi-target transformer excluding MSI) were lowest in the WHI cohort (0⋅77 [SD 0⋅01] *vs* 0⋅76 [0⋅01]; p=0⋅13) and highest in the CRA cohort (0⋅83 [0⋅02] *vs* 0⋅82 [0⋅03]; p=0⋅14); appendix pp 9–16, 32–33). In the detection of a *RNF43* mutation, mean AUROC was 0⋅80 (SD 0⋅05) for the single-target transformer and 0⋅86 (0⋅01; p=0⋅0021) for the multi-target transformer (figure 3A; appendix p 8). Mean AUROCs for *RNF43* comparing the primary model with the secondary model ranged from 0⋅80 (SD 0⋅01) versus 0⋅79 (0⋅01; p=0⋅77) in the TCGA cohort to 0⋅87 (0⋅02) versus 0⋅85 (0⋅01; p=0⋅061) in the CRA cohort (appendix pp 9–16, 32–33). For the detection of a *KRAS* mutation, mean AUROC was 0⋅65 (SD 0⋅02) for the single-target transformer and 0⋅65 (0⋅03) for the multi-target transformer (p=0⋅56; figure 3A; appendix p 8). Mean AUROCs for *KRAS* comparing the primary model with the secondary model ranged from 0⋅56 (0⋅02) versus 0⋅55 (0⋅02; p=0⋅16) in the TCGA cohort to 0⋅69 (0⋅06) versus 0⋅72 (0⋅03; p=0⋅39) in the CPTAC cohort (appendix pp 9–16, 32–33). No cases of MSI with *KRAS* mutation were observed in the CPTAC cohort, whereas up to 19 instances were reported in the TCGA cohort. The detection of hypermutation, *TP53* mutation, and *APC* mutation showed no significant differences between models (figure 3A; appendix p 8). Additional prediction targets with AUROCs of at least 0⋅75, including *BMPR2* and *ZNRF3* (figure 3A), are detailed in the appendix (pp 8–16, 32–33).

**Figure 4:**
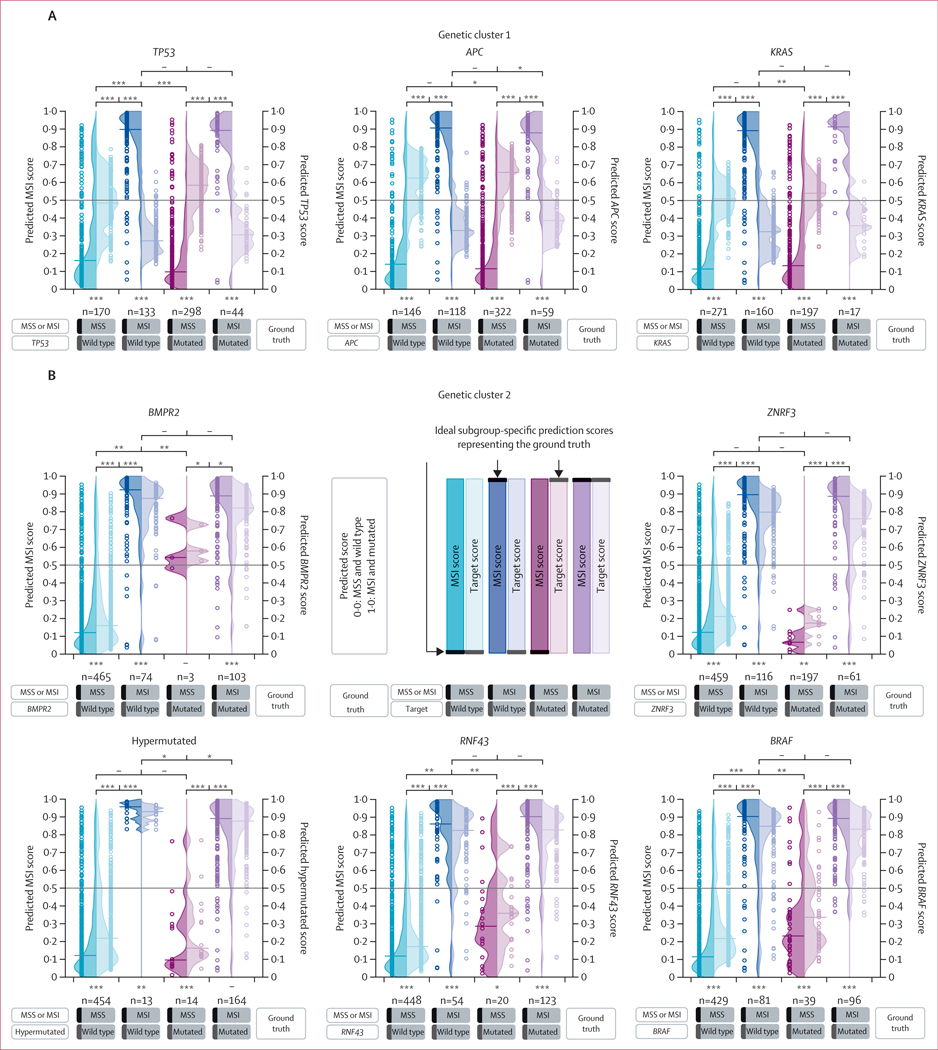
Evaluation of prediction scores based on the multi-target transformer in the external validation of the GECCO test set, subgrouped by the co-occurrence of prediction targets with MSI Violin plots representing individual patient scores from the test set cohorts for MSI and representative genetic alterations in four subgroups based on microsatellite status and mutational status. The left y axis represents the MSI score scale (left violin halves) and the right y axis corresponds to the prediction target scores (right violin halves). The legend shows grey horizontal lines in the concept violins that represent the optimal position of the prediction scores based on ground truth. (A) Genetic alterations predominantly co-occurring with MSS: *TP53*, *APC*, and *KRAS* (genetic cluster 1). (B) Genetic alterations predominantly co-occurring with MSI: *BMPR2*, *ZNRF3*, hypermutated, *RNF43*, and *BRAF* (genetic cluster 2). The data encompass both the CRA and WHI external cohorts. Each dot represents the mean value of individual patient prediction scores calculated from seven folds, with the horizontal line on each side of the violin indicating the median of all individual mean patient scores. A horizontal line at 0⋅50 denotes the line of model uncertainty. The sample count for each subgroup is indicated below the violins. Statistical significance is denoted as follows: *p<0⋅05, **p<0⋅01, and ***p<0⋅001. After testing for normal distribution (appendix p 30), the Mann–Whitney test was used for comparisons within groups and the Wilcoxon test was used for comparisons between groups. GECCO=Genetics and Epidemiology of Colorectal Cancer Consortium. MSI=microsatellite instability. MSS=microsatellite stability

Hierarchical clustering identified two primary genetic clusters. Cluster 1 included genes associated with MSS, such as *TP53*, *KRAS*, and *APC*; cluster 2 comprised genes associated with MSI (eg, *BRAF*, *BMPR2*, *ZNRF3*, and *RNF43*), with strong co-occurrence with MSI and hypermutation (figure 2). Association rule mining reinforced robust MSI correlations in cluster 2 (eg, *BMPR2* and *RNF43*), contrasting with the observation of inverse relationships in cluster 1 genes linked to MSS (eg, *TP53* and *KRAS*; appendix pp 17–19, 34–35). Cluster 2 showed higher AUROCs for mutation prediction than did cluster 1 (figure 3B, C; appendix pp 20, 31). Additional metrics and prediction score distributions (figures 1D, 3B, 4; appendix pp 20–25, 34–35) quantified model certainty, with prediction scores ranging from 0⋅00 (wild type, low) to 1⋅00 (mutated, high). Comprehensive summaries supported both internal and external validation (appendix pp 36–39).

For cluster 1 genes (*TP53*, *APC*, and *KRAS*), AUROCs for external validation ranged from 0⋅65 to 0⋅72, with MSI scores effectively distinguishing between cases of MSS and MSI (figures 2, 4A; appendix p 8). High MSI scores generally aligned with wild-type target predictions and low scores (MSS) with mutated predictions. Consequently, in MSI and mutated subgroups, as well as in MSS and wild-type subgroups, target predictions deviated from the ground truth. Cluster 2 genes (*BMPR2*, *ZNRF3*, *RNF43*, and *BRAF*) showed high AUROCs (0⋅75–0⋅88) for external validation (figures 2, 4B; appendix p 8). Alteration scores correlated with MSI scores across subgroups, yielding accurate trends in MSS and wild-type subgroups, as well as MSI and mutated subgroups, but deviating from the mutational ground truth in MSS and mutated subgroups and MSI and wild-type subgroups. These findings, partially reflected in AUROCs (figure 3B; appendix pp 34–35), indicate reduced mutated–wild-type differentiation in MSS and MSI subgroups relative to the combined group. In patients with MSS, mutations in *BMPR2* (three cases) and *ZNRF3* (nine cases) were rare; *RNF43* (20 cases), *BRAF* (39 cases), and *TP53* (298 cases) showed modest mutated– wild-type score separation (figure 4A, B), with slightly higher scores for mutated cases than wild-type cases suggesting partial differentiation. Although morphology associated with MSI was a pronounced factor in predicting phenotypes, aligning alteration-specific scores with MSS (cluster 1) or MSI (cluster 2) profiles, AUROCs of 0⋅60–0⋅70 and intermediate prediction scores in patients with MSS indicated minimal discrimination while suggesting that the model captured subtle phenotypic patterns (figure 3B; appendix pp 26–27, 34–35). Excluding MSI as a target led to mostly modest, mutation-specific performance shifts. The CRA cohort served as a representative example, in which *KRAS* showed increased sensitivity at the expense of specificity, whereas *BRAF* remained stable (appendix pp 9, 13).

**Figure 5:**
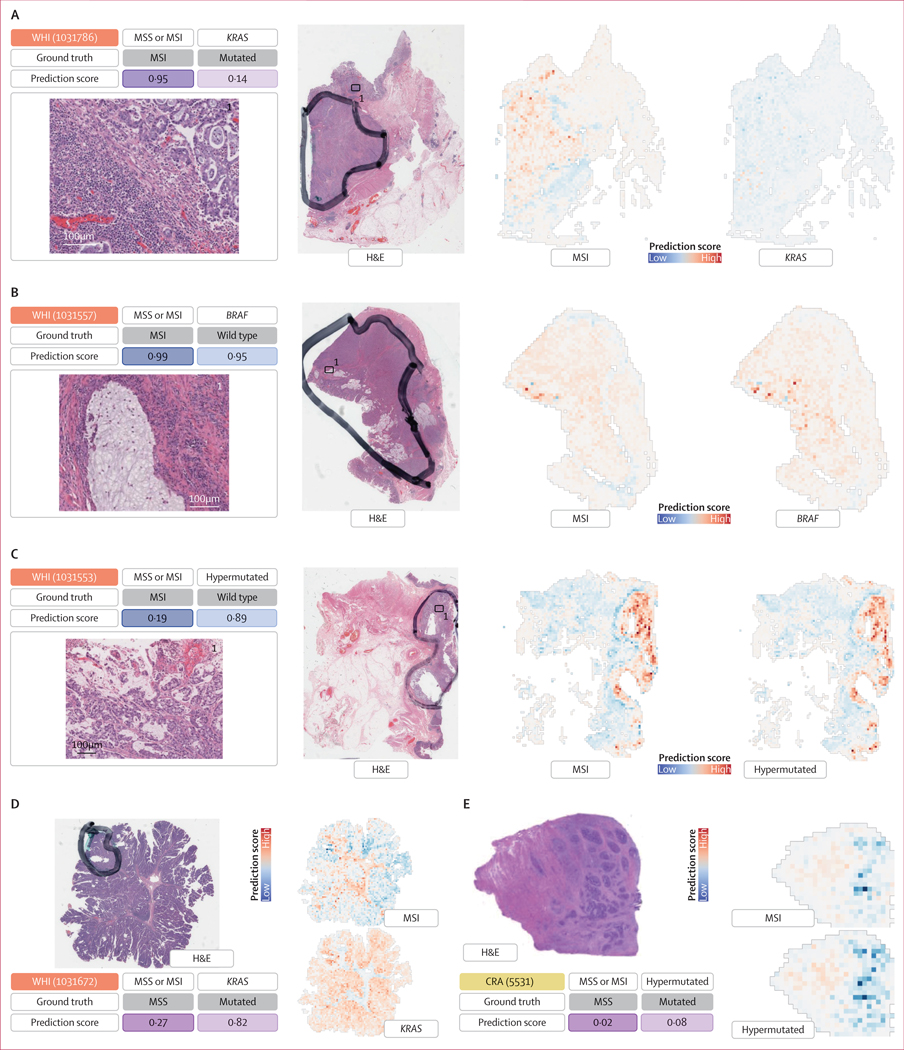
Heatmaps of representative samples for the prediction of MSI, *KRAS, BRAF*, and hypermutated status from the external GECCO validation dataset The heatmaps are derived from the model with median AUROC for the detection of MSI and most prediction targets evaluated by seven-fold cross-validation. The cohort, sample ID, ground truth, and prediction scores for MSI, along with the individual mutational status of the target and magnified views of specific areas are provided for in-depth analysis. The heatmaps indicate relevant areas for the various predictions. The red areas indicate a high score or mutant type, whereas the blue areas indicate a low score or wild type. The colour intensity demonstrates the model’s attention to that distinct area. Pen markings denote regions annotated by various pathologists, highlighting tumour or other areas of interest. The meaning and consistency of these annotations vary, given that no uniform annotation protocol was applied. (A) The tumour shows both gland-forming and more solid components, as well as extremely high numbers of tumour-infiltrating lymphocytes with dense lymphoid aggregates. The pathological examination confirms the plausibility of a high MSI score indicating MSI, which is also the ground truth. A low *KRAS* score indicates wild-type *KRAS* but the ground truth is mutated *KRAS*. The heatmap highlights similar tumour areas but with diverging scores: where the MSI map is red indicating a high score, the *KRAS* map is blue indicating a low score. (B) The presence of mucinous differentiation in MSI. Wild-type *BRAF* results in high MSI and *BRAF* scores. The MSI score is pathologically plausible, whereas the *BRAF* score indicates a contrary prediction tendency compared with the ground truth. For both predictions, the model focuses on similar tumour areas with similar scores, indicating MSI or mutated *BRAF*. (C) Partly mucinous morphology indicates the possibility of MSI, with a high score predicting MSI. Hypermutation is also predicted with a high score, even though hypermutation is not present for this sample. Both heatmaps primarily label the tumour and the same region with similar relevance. (D) Villous adenoma with high-grade dysplasia is a common precursor lesion associated with a high frequency of *KRAS* mutations.^27,28^ The heatmaps highlight similar large-scale tumour areas but with converging scores; where the MSI map is red indicating a high score, the *KRAS* map is blue indicating a low score. (E) The tumour area seems to show mainly MSS and the heatmap predicts a correspondingly low score. Although the tumour seems hypermutated in the ground truth, its prediction score indicates non-hypermutated. This tumour is a rare MSS case with hypermutation. Both heatmaps predominantly mark the tumour area and the same region with similar relevance. AUROC=area under the receiver operating characteristic curve. CRA=Cancer Risk Assessment study. GECCO=Genetics and Epidemiology of Colorectal Cancer Consortium. H&E=haematoxylin and eosin. MSI=microsatellite instability. MSS=microsatellite stability. WHI=Women’s Health Initiative.

**Figure 6:**
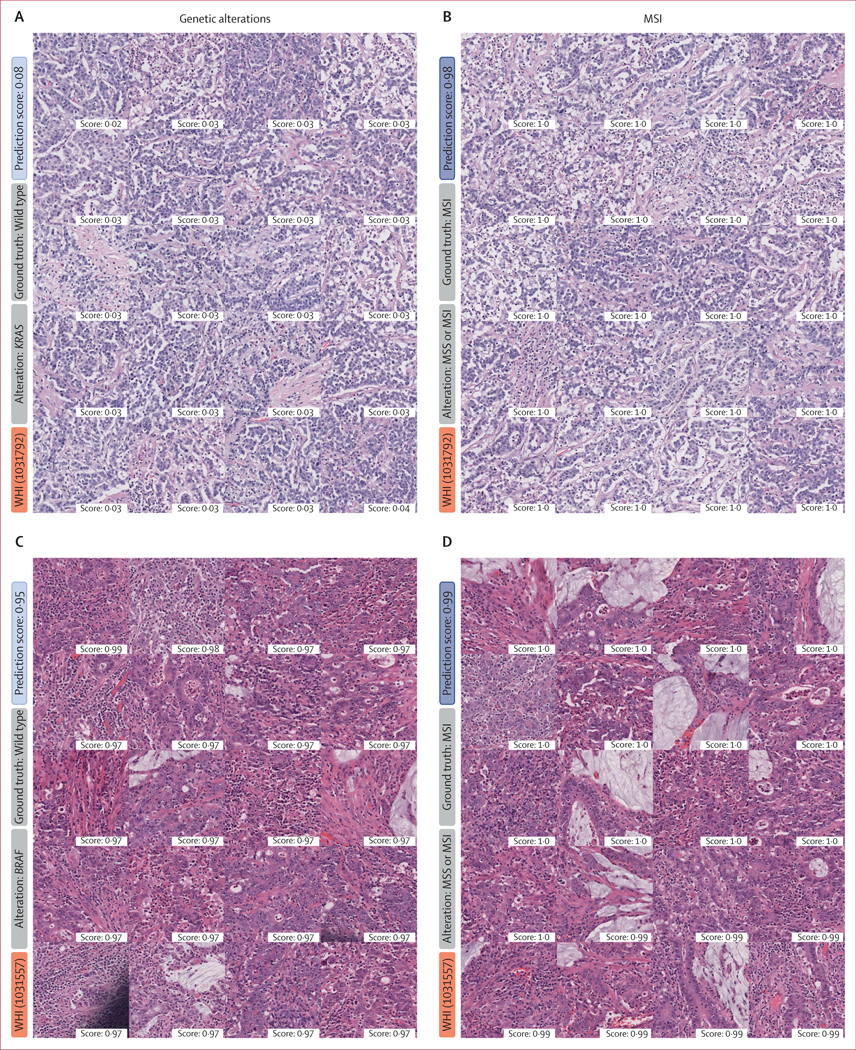
Top tiles for the prediction of genetic alterations and MSI for two selected slides from the GECCO test set (A–B) WHI (1031792): medullary carcinoma with sheets of tumour cells, low stroma content, and high number of tumour-infiltrating lymphocytes in a wild-type *KRAS* and MSI case, leading to low mutated *KRAS* prediction scores (A) and high MSI prediction scores (B). Colorectal cancers with MSI show reduced frequency of *KRAS* mutations. Medullary carcinoma is a key morphological feature of colorectal cancers with MSI. (C–D) WHI (1031557): top tiles for the prediction of mutated *BRAF* and MSI. Both predictions have high prediction scores and show a mixed morphology with partly medullary, mucinous, and gland-forming histology, as well as a high number of tumour-infiltrating or associated lymphocytes. Medullary growth pattern with lymphocytic infiltration and mucinous differentiation are typical features of an MSI-like morphology. Accordingly, the case was correctly predicted as MSI with a high prediction score (D). Given that mutated *BRAF* and MSI often co-occur and share morphological overlap, the case was misclassified with regard to *BRAF* status, resulting in high prediction scores for mutated *BRAF* (C), even though the ground truth was wild-type *BRAF*. GECCO=Genetics and Epidemiology of Colorectal Cancer Consortium. MSI=microsatellite instability. MSS=microsatellite stability. WHI=Women’s Health Initiative

To investigate underlying morphological patterns of predicted genetic alterations, we manually reviewed whole-slide image heatmaps and the top 20 high-attention tiles per target across 25 cases from the CRA and WHI cohorts (figures 5, 6; appendix pp 28–29, 40–62). Targets included key alterations stratified by cluster: *TP53*, *APC*, *KRAS* in cluster 1; and *RNF43*, *BRAF*, and hypermutation in cluster 2. Model attention was predominantly directed toward tumour regions, with minimal attribution to pen marks or non-tumour areas (figure 5A–D; appendix pp 40–43), despite the absence of explicit tumour labels during training. Although pen marks were present on most slides, they were rarely highlighted (figure 5C; appendix pp 46–47) and appeared faintly in top tiles (appendix pp 52, 61).

Frequent features associated with MSI, including medullary growth, high number of tumour-infiltrating lymphocytes, and mucinous differentiation, were observed in most MSI and cluster 2 gene mutations. However, some cases of MSI showed atypical morphologies (appendix pp 44–47). In cluster 1, predictions of *KRAS* mutations assigned high attention to luminal tumour regions, particularly villous adenomas with high-grade dysplasia and invasive adenocarcinoma (appendix pp 55–56), reflecting the model’s capability to capture heterogeneity within tumours. Tiles with high MSI relevance showed medullary carcinoma features, including tumour cell sheets and a high number of tumour-infiltrating lymphocytes, and yielded low *KRAS* prediction scores (figure 6A, B). Alterations in *TP53* and *APC*, less frequent in MSI than in MSS, were associated with MSS-like morphology, including gland-forming adenocarcinoma and dirty necrosis (appendix pp 57–59). Alterations linked to MSI, such as *BRAF*, *RNF43*, and hypermutation, were associated with medullary patterns, mucinous differentiation with signet-ring cells (appendix p 62), and a high number of tumour-infiltrating lymphocytes (figure 6C–D; appendix pp 28–29, 51–54, 60), all features characteristic of MSI. The pathological review highlighted tumour budding as a potential morphological correlate of *BRAF* mutations, particularly in MSS cases (appendix p 62).

## Discussion

Many studies have used deep learning to predict biomarkers from pathology slides, leading to clinically approved or evaluated tools. However, these studies typically focus on a single target, such as MSI in colorectal cancer, homologous recombination deficiency in breast cancer,^21^ or *EGFR* in lung cancer.^22^ In this study, we address this limitation by predicting various biomarkers and analysing their interactions and shared histological patterns in colorectal cancer. By use of five GECCO cohorts with harmonised sequencing and external validation in the TCGA and CPTAC cohorts, the multi-target model achieved performance within the literature range, out-performing single-target models for some alterations while enabling efficient and scalable prediction of multiple genetic targets. The cohorts, primarily drawn from the USA and Europe, were broadly representative of North American and European patient populations with colorectal cancer.^23^ The IWHS and WHI cohorts were predominantly White and increased the representation of female patients; the TCGA cohort included the most Black or African American patients; and CPTAC, limited to fresh−frozen samples, was the smallest cohort. MSI frequency ranged from 7% to 35%, consistent with reported rates (15–20%),^24^ and mutation frequencies for key genes (ie, *TP53*, *APC*, *KRAS*, and *BRAF*)^3,4,7–9^ aligned with previous studies.^17^

Consistent with previous studies,^3–5,8,9,20^ MSI was the most reliably predicted alteration; however, some cases scored low due to the absence of typical morphological features,^25^ which was confirmed pathologically. Additionally, some cases of MSI showed atypical morphologies not commonly linked to MSI.^26^ Predictions of *KRAS* mutations assigning high attention to luminal tumour regions, particularly villous adenomas with high-grade dysplasia and invasive adenocarcinoma, are consistent with known associations of *KRAS* mutations with villous adenomas^27^ and tumours adjacent to polyps.^28^ For MSI detection, the mean range of AUROCs for the primary model was consistent with the range of 0⋅77–0⋅96 reported in the literature.^2–6,8,9,20^ Mean AUROCs for *BRAF* in the primary model also aligned with those reported in the literature (0⋅66 to 0⋅88).^3–10,20^ Mean AUROCs for *RNF43* comparing the primary model with the secondary model (0⋅80 *vs* 0⋅79 in the TCGA cohort to 0⋅87 *vs* 0⋅85 in the CRA cohort) exceed the literature range of 0⋅63 to 0⋅72.^7,10^ Mean AUROCs for *KRAS* comparing the primary model with the secondary model (0⋅56 *vs* 0⋅55 in the TCGA cohort to 0⋅69 *vs* 0⋅72 in the CPTAC cohort) fall below and within the literature range of 0⋅60 to 0⋅80.^3,5,7,8,10,20^

Results for the detection of hypermutation fell within the literature range of 0⋅81–0⋅87,^3,6,9^ as did those for *TP53* mutation: 0⋅60–0⋅75.^3,6,7,10^ For the prediction of *APC* mutational status, the results ranged above and below the single available literature AUROC of 0⋅67.^7,10^

For several mutations, our multi-target model out-performed single-target baselines and published models. Prediction of hypermutation showed strong association with MSI due to their frequent co-occurrence and shared morphological features, which lowered scores in cases of hypermutated MSS. Excluding MSI as a target led to mostly modest, mutation-specific performance changes, consistent with largely independent class token behaviour and limited cross-token interaction in the decoder (appendix p 63). Given the presence of morphology associated with MSI in whole-slide images, features shared with other alterations, such as *BRAF*, might have influenced predictions. *BRAF* mutations showed detectable phenotypic changes, including mucinous differentiation and poorly differentiated clusters.^29^ Histopathology confirmed mucinous patterns predominated in mutated *BRAF* tiles and stroma-rich patterns predominated in wild-type *BRAF* tiles. Mucinous or signet-ring features contributed to *BRAF* prediction but overlapped with morphology associated with MSI, causing misclassifications in MSS.^8,29^ Some mutated *BRAF* tiles showed poorly differentiated clusters and tumour budding, linked to *BRAF* mutations and MSS.^30^ MSI in colorectal cancer shows distinct histopathological features, such as medullary growth, mucinous differentiation, and prominent tumour-infiltrating lymphocytes, which support its high predictive performance in models based on deep learning. These features frequently co-occur with mutations in *BRAF* and *RNF43*, suggesting that predictions are influenced by shared morphology. Morphology associated with MSI reflects a composite phenotype closely linked to MSI and co-occurring mutations (eg, *BRAF*). This dominant morphology, linked to diverse yet intertwined alterations, drives predictions but could mask subtler, mutation-specific patterns. This morphology functions as an integrated phenotype rather than a confounder and influences targets differentially.

This study has several limitations. Despite the dataset’s considerable scope, the detection of rare mutations and their associated subtle morphologies showed variable performance, likely due to the small sample sizes of these alterations. Under-representation of non-White individuals and missing annotations further limit generalisability and accuracy, highlighting the need for even larger, more diverse cohorts. Although infrequent and faint, residual pen markings occasionally attracted model attention, potentially introducing minor bias or capturing features near tumour margins, warranting further investigation. These findings emphasise the need for more advanced explainability methods to validate patterns derived from deep learning and link them to specific molecular targets, enabling reciprocal insights between conventional and computational pathology. Such methods might clarify how morphology associated with MSI obscures subtler mutation-specific features, a dominance that could be mitigated by multimodal integration or unlearning strategies to reveal less overt morphologies. Our model, extending single-target transformers, might exhibit bias toward targets associated with MSI due to a greater number of co-occurring mutations with MSI than with MSS, warranting further investigation of model mechanisms. Given that AUROCs alone can misrepresent the predictability of biomarkers, evaluation should include additional metrics, individual scores, a co-occurrence analysis, and a pathological review. Additionally, future studies should use diverse datasets, evaluate multiple targets across multiple metrics, and address overlapping morphologies through approaches such as our multi-target model.

Our framework offers practical value by identifying predictable targets and their morphological basis; follow-up studies could spatially correlate predictions based on deep learning with existing molecular assays, such as surrogate immunohistochemistry for *BRAF* alterations,^31^ supporting explainability and elucidating underlying biological mechanisms. Clinically, this scalable approach streamlines diagnostics by enabling low-cost pre-screening, particularly for patients with early-stage colorectal cancer and in resource-limited settings. Biologically, this approach illustrates how genomic alterations shape morphology and reinforces the role of surrogate markers, such as MSI, in genotype−phenotype interplay. Given that co-occurrence analysis remains underexplored in studies based on deep learning, particularly beyond colorectal cancer,^32^ this approach enables efficient, simultaneous prediction of multiple targets across cancers, supporting broader precision-oncology research.

In conclusion, multitarget transformers enable efficient, simultaneous prediction of biomarkers and investigation of biomarker-specific patterns from histopathology slides stained with haematoxylin and eosin, with morphology associated with MSI emerging as the dominant predictive feature in colorectal cancer. The model’s reliance on shared and distinct histological patterns across alterations highlights the limitations of AUROCs and underscores the need for a subgroup-based analysis incorporating both morphological context and the co-occurrence of alterations. Importantly, future studies based on deep learning should account for shared phenotypes, and past results should be interpreted accordingly. This approach advances the integration of computational pathology, establishing a foundation for broader application across cancer types.

## Supplementary Material

Supplementary Material

## Data Availability

All source code used to conduct this study is publicly available with publication. The code for image preprocessing is publicly available at https://github.com/KatherLab. The tessellation script is available at https://github.com/KatherLab/preprocessing-ng. Extraction of CTransPath features was conducted with scripts from https://github.com/KatherLab/marugoto. The scripts for the multi-target transformer, heatmap generation, and top tile extraction for explainability, as well as the saved checkpoints for the trained models with the lowest validation loss across all seven folds, are available at https://github.com/KatherLab/MultiTargetCRC. Links to the exact repository versions used can be found in the appendix (p 7). The molecular and clinical data for the TCGA and CPTAC cohorts, as well as the digitised whole-slide images for TCGA,^13^ are publicly accessible at the Genomic Data Commons Data Portal and cBioPortal for Cancer Genomics. The digitised whole-slide images for CPTAC are publicly accessible at https://www.cancerimagingarchive.net/collection/cptac-coad/.^16^ The datasets from the GECCO consortium are available from the corresponding author on reasonable request. All data generated or analysed during this study are included in this Article and the appendix.

## Research in context

### Evidence before this study

To assess the current landscape of research on artificial intelligence in colorectal cancer histopathology, we performed a comprehensive literature search. On April 18, 2024, we searched PubMed, without restrictions on language or date, for original research and review articles using the following search terms: (“colorectal cancer” OR “colon cancer” OR “rectal cancer”) AND (“deep learning” OR “machine learning” OR “artificial intelligence”) AND (“mutation” OR “microsatellite” OR “molecular”) AND (“histopathology” OR “pathology” OR “histology” OR “HE” OR “H&E” OR “WSI” OR “digitized slides” OR “whole slide images”). We found 262 studies, many of which showed that deep learning can predict molecular alterations (eg, mutations, microsatellite instability [MSI], or both) directly from histopathological slides stained with haematoxylin and eosin from patients with colorectal cancer. These previous studies relied on a single-target prediction architecture. Even though some studies report that the accuracy of predictions might decrease within some subpopulations (eg, *BRAF*-mutant colorectal cancers), none of these studies have systematically accounted for the potential confounding effects of co-occurring molecular alterations and shared morphology.

### Added value of this study

In this multicentre cohort study, we used slides stained with haematoxylin and eosin from 1912 patients with colorectal cancer across seven independent cohorts, including two public datasets. We developed a multi-target transformer model capable of simultaneously predicting several genetic alterations from the slides. Our multi-target model matched and partly exceeded established single-target models in predicting numerous biomarkers, such as *BRAF* or *RNF43* mutations and MSI, from pathology slides. Interestingly, biomarkers with high predictability (eg, area under the receiver operating characteristic curve >0⋅7) were strongly correlated with MSI, which is known to be associated with distinct morphological features detectable with deep learning algorithms. The dominance of morphology associated with MSI in predictions challenges the assumption that multiple genetic biomarkers can be independently predicted, suggesting instead that co-occurring alterations are inferred through shared morphological features, thereby advancing understanding of morphological genomic interplay in colorectal cancer.

### Implications of all the available evidence

Our study shows the value of multi-target transformers for predicting multiple genetic alterations from slides stained with haematoxylin and eosin of colorectal cancer, although these predictions were often linked to morphology associated with MSI. These findings highlight the influence of cancer-specific phenotypic and genetic co-occurrences, underscoring the need to account for their impact in both past and future studies evaluating deep learning models for potential clinical integration.
